# Patient's View on: “Changes in body composition and handgrip strength during dapagliflozin administration in patients with chronic kidney disease”^*^

**DOI:** 10.1093/ckj/sfaf121

**Published:** 2025-04-18

**Authors:** Takamasa Iwakura

**Affiliations:** First Department of Medicine, Division of Nephrology, Hamamatsu University School of Medicine Hamamatsu, Japan

Sodium-glucose cotransporter 2 (SGLT2) inhibitors are increasingly being prescribed for patients with chronic kidney disease (CKD) and chronic heart failure. In older adults, CKD is often associated with atherosclerotic disease, and heart failure is also frequently observed. In these populations, one of the key concerns when prescribing SGLT2 inhibitors is the potential for long-term skeletal muscle loss and the progression of sarcopenia.

This study's finding—that the prevalence of sarcopenia did not increase over a 24-week period—is both interesting and somewhat reassuring. However, from a patient's perspective, I strongly feel that it is essential to understand the long-term effects. SGLT2 inhibitors are often taken continuously over years, with the aim of preserving kidney function and protecting the heart. For patients to feel fully confident in taking them, we need more research into their prolonged impact on muscle mass and sarcopenia.

In fact, the data in this study show a gradual, statistically significant decrease in Skeletal Muscle Mass Index (SMI) over 24 weeks. This raises a concern about whether this decrease will stabilize over time or continue progressively. From the patient's point of view, what matters most is not only the muscle quantity but also its effect on quality of life. In elderly patients, even modest muscle loss can affect the ability to walk or perform daily activities, or increase the risk of falls due to leg weakness.

Finally, I hope future research will focus on establishing preventive strategies to mitigate muscle loss associated with SGLT2 inhibitors. Doing so would allow patients to fully benefit from the organ-protective effects of these medications, without compromising mobility or independence.

